# Expression Differences of Pigment Structural Genes and Transcription Factors Explain Flesh Coloration in Three Contrasting Kiwifruit Cultivars

**DOI:** 10.3389/fpls.2017.01507

**Published:** 2017-09-01

**Authors:** Yanfei Liu, Bin Zhou, Yingwei Qi, Xin Chen, Cuihua Liu, Zhande Liu, Xiaolin Ren

**Affiliations:** ^1^College of Horticulture, Northwest A&F University Yangling, China; ^2^Shaanxi Fruit Industry Group Yangling, China

**Keywords:** expression differences, structural genes, transcription factors, color diversity, kiwifruit

## Abstract

Fruits of kiwifruit cultivars (*Actinidia chinensis* and *A. deliciosa*) generally have green or yellow flesh when ripe. A small number of genotypes have red flesh but this coloration is usually restricted to the inner pericarp. Three kiwifruit cultivars having red (‘Hongyang’), or yellow (‘Jinnong-2’), or green (‘Hayward’) flesh were investigated for their color characteristics and pigment contents during development and ripening. The results show the yellow of the ‘Jinnong-2’ fruit is due to the combined effects of chlorophyll degradation and of beta-carotene accumulation. The red inner pericarps of ‘Hongyang’ fruit are due to anthocyanin accumulation. Expression differences of the pathway genes in the inner pericarps of the three different kiwifruits suggest that *stay-green* (*SGR)* controls the degradation of chlorophylls, while *lycopene beta-cyclase* (*LCY-*β*)* controls the biosynthesis of beta-carotene. The abundance of anthocyanin in the inner pericarps of the ‘Hongyang’ fruit is the results of high expressions of *UDP flavonoid glycosyltransferases* (*UFGT)*. At the same time, expressions of anthocyanin transcription factors show that *AcMYBF110* expression parallels changes in anthocyanin concentration, so seems to be a key R2R3 MYB, regulating anthocyanin biosynthesis. Further, transient color assays reveal that *AcMYBF110* can autonomously induce anthocyanin accumulation in *Nicotiana tabacum* leaves by activating the transcription of *dihydroflavonol 4-reductase* (*NtDFR*), *anthocyanidin synthase* (*NtANS)* and *NtUFGT*. For basic helix-loop-helix proteins (bHLHs) and WD-repeat proteins (WD40s), expression differences show these may depend on *AcMYBF110* forming a MYB-bHLH-WD40 complex to regulate anthocyanin biosynthesis, instead of it having a direct involvement.

## Introduction

Kiwifruit (*Actinidia*, *Actinidiaceae*) is a dioecious, deciduous, perennial plant with a climbing growth habit (Li et al., 2007). It has long been called the ‘king of fruits’ due to its unique flavor and exceptional nutritional value. Its nutritional components include: abundant vitamin C, amino acids, balanced mineral composition, high dietary fiber and various other healthful metabolites (Chen et al., 2013).

According to Li et al. (2007), *Actinidia* comprises around 75 distinct species, with their multi-seeded fruits (berries) exhibiting a wide range of skin and flesh colors. This diversity is used by breeders to produce novel kiwifruit cultivars which will help it to further increase its market share, while also increasing its health benefits. Two economically important species, *Actinidia chinensis* and *A. deliciosa*, have dominated international trade because of their large-fruit, unique flavors and good storage properties (Chen et al., 2013). In recent years, because of the brilliant colors and rich functional components (phenols, flavonoids and carotenoids, etc.) of the yellow- and red-fleshed kiwifruits, increasing international market and consumer attention are being paid to these, compared with to their green-fleshed cousins which have tended to monopolize the market till now. Therefore, the coloring mechanisms of the yellow- and red-fleshed kiwifruit have become a special focus for increasing numbers of researchers.

Chlorophyll, carotenoids and anthocyanins are the most important color pigments of plant tissues, including fruit. In most plant tissues, the bright yellow colors are caused by increases in carotenoid content, such as in pepper (Guzman et al., 2010), carrots (Clotault et al., 2008) and sweet oranges (Zeng et al., 2015). However, for kiwifruit, Montefiori et al. (2009b) suggested the yellow color of yellow-fleshed kiwifruit was caused by a disappearance of the chlorophylls, rather than by an increase in the carotenoids. This process is controlled by the expression level of *SGR2* (Pilkington et al., 2012). Ampomah-Dwamena et al. (2009) reported that the concentration of beta-carotene, the major carotenoid in the three yellow-fleshed kiwifruit species tested, increases rapidly during maturation and ripening. Moreover, the increase in beta-carotene seems to be positively controlled by the expression level of *LCY-*β (Ampomah-Dwamena et al., 2009). Hence, it is speculated that the yellow color formation is controlled by both chlorophyll-related genes and by carotenoid-related genes. Carotenoids also contribute to the red color formation in a few fruit species, typically in red citrus (Zeng et al., 2015), red loquat (Fu et al., 2012) and red pepper (Tian et al., 2014). While for most plants, such as apple, pear and peach, the red color is caused by the presence of anthocyanins (Telias et al., 2011; Wang et al., 2013; Zhou et al., 2015). Hence, kiwifruit is not exceptional (Montefiori et al., 2009a, 2011; Man et al., 2015). That anthocyanins, deriving from the flavonoid branch of the phenylpropanoid metabolic pathway, has been extensively studied in many fruit species (El-Sharkawy et al., 2015; Cho et al., 2016). The most important transcription factors are MYBs, bHLHs and WD40s, which regulate the downstream enzyme genes by forming the MYB-bHLH-WD40 (MBW) complex (Schaart et al., 2013; Xu et al., 2014, 2015). In kiwifruit, most enzyme genes controlling anthocyanin biosynthesis have already been reported (Li et al., 2007, 2015; Yang et al., 2009, 2010; Montefiori et al., 2011; Zhang et al., 2012). However, there are few studies to reveal the molecular mechanism of transcription factors regulate anthocyanin biosynthesis and accumulation in flesh of red-fleshed kiwifruit (Man et al., 2012, 2015; Li et al., 2015, 2017). *AcMYB110* is an R2R3 MYB regulating the coloration of the red petals in kiwifruit (Fraser et al., 2013). This shows strong activation of the anthocyanin pathway in *Nicotiana tabacum* leaves (Montefiori et al., 2015). However, its expression product is undetectable in the fruit of red-fleshed kiwifruit (Li et al., 2015).

To investigate the molecular mechanisms of color differences in different-colored kiwifruits, three representative kiwifruit cultivars were selected. These were: ‘Hongyang,’ one of the main commercial kiwifruit cultivars now grown in China, contains high concentrations of anthocyanin. These anthocyanins accumulate mainly in the inner pericarp, creating an attractive, red, star-shape in the center; ‘Jinnong-2,’ because of its yellow flesh, high fruit quality and yield, and great potential in stress resistance, is being widely promoted in Shannxi province of China; ‘Hayward,’ as is known to all, has been widely cultivared around the world, therefore it is a good material as a green control for research of kiwifruit coloration. We identified the key genes controlling the development of yellow and red coloration in kiwifruit flesh by detecting the concentrations of chlorophyll, carotenoids and anthocyanin in the outer and inner pericarps of fruit. Also, by recording the differential structural genes and by carrying out transcription factor expression analyses at different developmental stages. These findings help to create a firm theoretical basis for further study of the molecular mechanisms of color difference in different-colored kiwifruit. They are also valuable for plant breeders, given the challenges of developing new kiwifruit varieties from within a genus.

## Materials and Methods

### Fruit Materials

Three kiwifruit cultivars of contrasting coloration were selected (**Table 1**). Sampling dates for each cultivar are reported as days after pollination (DAP). Samples were taken about every 20 days (Supplementary Table S1). At each sampling time, 20 fruits were collected at random from three vines of each of the three cultivars. Fruits were collected of each genotype at seven preharvest stages: on 25, 45, 65, 85, 105, 125, and 145 DAP. ‘Hongyang’ and ‘Hayward’ are commercial cultivars in China and their times of harvest were determined according to the normal industry criteria: ‘Hongyang,’ when the soluble solids content (SSC) was ≥7.0° Brix, and ‘Hayward,’ when the SSC was ≥6.5° Brix. Meanwhile, ‘Jinnong-2’ is a commercial cultivar in Shaanxi province but not widely grown outside China. We set its commercial harvest time as being when the SSC ≥ 10.0° Brix because this criterion is commonly used for screening breeding populations of *A. chinensis*. For a long time, the dry matter content of ≥15.0% has been a industry criterion for determining harvest time for kiwifruit. By 145 DAP, the fruits of all three cultivar had reached commercial harvest maturity (**Table 1**). Fruits were then harvested and stored at room temperature (c. 22°C). A total of 20 fruits of each genotype were examined after 10 days storage (155 DAP) and a further 20 fruits at full ripeness – fruit firmness was about 9.8 N on 170 DAP (‘Hongyang’ and ‘Jinnong-2’) or on 190 DAP (‘Hayward’).

**Table 1:** Cultivars, color, soluble solid content, dry matter content and firmness of kiwifruits.

Cultivars	Species	Ripe fruit color		Soluble solid content (° Brix)		Dry matter content (%)		Firmness (N)	
		Outer pericarp	Inner pericarp	Harvest	Full ripe	Harvest	Full ripe	Harvest	Full ripe
Hongyang (HY)	*A. chinensis*	Yellow-green	Red	7.30 ± 0.15	21.17 ± 0.74	18.39 ± 1.41	26.02 ± 0.12	49.78 ± 0.74	9.79 ± 1.08
Jinnong-2 (JN)	*A. chinensis*	Yellow	Dark yellow	11.40 ± 0.81	18.27 ± 0.19	18.21 ± 0.11	20.10 ± 0.26	40.67 ± 0.59	9.40 ± 0.15
Hayward (HWD)	*A. deliciosa*	Green	Green	6.63 ± 0.12	15.67 ± 0.35	18.74 ± 1.65	19.64 ± 1.16	55.39 ± 1.13	9.95 ± 0.15

Of the 20 fruits of each cultivar at each stage, 10 fruits were used to measure the various physiological parameters (i.e., SSC, dry matter content, firmness and color indices). After peeling (removing the skin to approximately 1 mm depth), the other 10 fruits were carefully separated into outer and inner pericarps (seeds were removed) and the separated tissues immediately frozen in liquid nitrogen. Petals, stems, leaves and ovaries were collected from vines of ‘Hongyang.’ Fruits of another three, red-fleshed cultivars (‘Qihong,’ ‘Donghong’ and ‘Purpurea’) and of three, green-fleshed cultivars (‘Xuxiang,’ ‘Cuixiang’ and ‘Jinkui’) were also collected at maturity. All samples were taken from the National Center of Kiwifruit Breeding, Mei County, Shannxi province, China. The tissues were frozen in liquid nitrogen and stored at -80°C pending analysis.

### Measurements of Fruit Firmness, Soluble Solids and Dry Matter Content

Fruit flesh firmness was determined by penetration at the two opposite cheeks of each fruit after removal peel (1 mm thick, 1 cm^2^) using the GUSS Fruit Texture Analyzer (GS-15, Strand, South Africa) with a 8 mm plunger (Minas et al., 2016). The same fruit was used to measure the SSC, expressed as ° Brix, by taking two juice samples from the equatorial part of each fruit and measuring with a hand-held refractometer (PAL-1, Atago, Japan). The 2 mm transversal slice came from the equator of each fruit was uesd to measure the dry matter content. The fruit slices after weighed their fresh weights (FW) were then dried at 105°C in a vacuum oven (DZF-6050, Shanghai, China) to constant weight (DW). The dry matter content is FW/DW ^∗^ 100% (Famiani et al., 2012). Three repeated were carried out for these physiological parameters.

### Color Measurement

The colors of the outer and inner pericarp tissues at the various stages were measured using a chroma meter (CR-400, Konica Minolta, Japan) based on the CIE L^∗^, a^∗^, b^∗^ mode (Hanbury and Serra, 2002). Five random measurements were carried out on the outer and inner pericarps of each fruit transverse section. The data are described as L^∗^ (lightness), a^∗^ (red-green sensation), b^∗^ (yellow-blue sensation) and h° [hue angle, tan^-1^(b^∗^/a^∗^)]. Three replicates were performed for each sample.

### Measurements of Chlorophyll, Carotenoid and Anthocyanin

Chlorophyll and carotenoids were extracted from the fruits following the method of Montefiori et al. (2009b), and quantified using the method of Tian et al. (2014). Three replicates were performed for each sample.

The anthocyanins were extracted with 1% (v/v) hydrochloric acid/methanol and held for 24 h at 4°C in the dark. The supernatant was filtered through a 0.22 μm syringe. Extracts were analyzed as previously described (Bi et al., 2014). Three replicates were performed for each sample.

### Expression Analysis by Quantitative Real-Time PCR (qPCR)

Total RNA was extracted using the Plant RNA Kit (Omega Bio-tek, Norcross, GA, United States). The RNA concentration and quality were determined by UV spectrophotometry and by running on a 1.0% agar ethidium bromide-stained gel. Approximately 1 μg of total RNA was used for cDNA synthesis with the PrimeScript RT reagent kit (TaKaRa, Dalian, China). Quantitative real time PCR was carried out with SYBR Premix ExTaq II Kit (TaKaRa, Dalian, China), and amplification was monitored on an Icycler iQ5 (Bio-Rad, Berkeley, CA, United States) in a reaction volume of 20 μL.

The amplification program consisted of one cycle of 95°C for 40 s followed by 40 cycles of 95°C for 30 s and 59°C for 30 s. Melting curve analysis was carried out after 40 cycles to ensure the proper amplification of target fragments. Actin was used for normalization. All analyses were repeated three times using biological replicates. Primer sequences are listed in Supplementary Table S2.

### Identification of Candidate Transcription Factors in Kiwifruit and a Phylogenetic Analyses

Using the sequences of MYB, bHLH and WD40 which have been identified to promote anthocyanin synthesis in other plants, we blasted the genome of kiwifruit^1^, resulting in five MYBs, five bHLH and two WD40 members being identified. The nucleotide sequences were used for phylogenetic analyses. Sequences were aligned using ClustalW and adjusted manually as necessary. The resulting data were analyzed by the Neighbor Joining method using the MEGA 6.0 program. They were named based on the results of the phylogenetic analyses.

### Isolation and Sequence Alignments of *AcMYBF110*

Full-length cDNAs of *AcMYBF110* were amplified from ‘Hongyang’ fruits by specific primers (given in Supplementary Table S2) based on the available sequences in the kiwifruit genome database^1^. The amino acid sequences encoded by *AcMYBF110* and anthocyanin-promoting MYBs from various plants were used for multiple alignment using DNAMAN software.

### Subcellular Localization of AcMYBF110

The coding region without stop codon of *AcMYBF110* with C-terminal GFP fusion was inserted into the multiple cloning site of plant binary expression vector pVBG2307 (Ahmed et al., 2012) to form 35S:AcMYBF110-GFP. The primers are shown in Supplementary Table S2. Vector which inserted only with the GFP gene (35S:GFP) was used as positive control. Two constructs were transformed to *Agrobacterium tumefaciens* strain GV3101 using a freeze-thaw method. Then the *Agrobacterium* strains were infiltrated into 6-week-old *Nicotiana benthamiana* leaves. After incubating at 24°C with 16 h light for 48–72 h, the fluorescence was observed and DAPI was used to locate the fluorescent proteins in the nucleus.

### Heterologous Overexpression of *AcMYBF110* in Tobacco

Six-week-old *N. tabacum* leaves were used for infiltration. The above strains containing 35S:AcMYBF110-GFP and control (35S:GFP) were infiltrated into the abaxial leaf surface. Each infiltration was carried out on three leaves. The infiltrated plants were placed in the dark at 24°C overnight and then transferred to a growth chamber at 24°C with a 16 h light/8 h dark cycle under low light conditions. Plants were photographed and sampled 6 days after being infiltrated. All samples were frozen in liquid nitrogen and stored at -80°C, pending analyses.

## Results

### Colors of the Three Kiwifruit Cultivars

**FIGURE 1:**
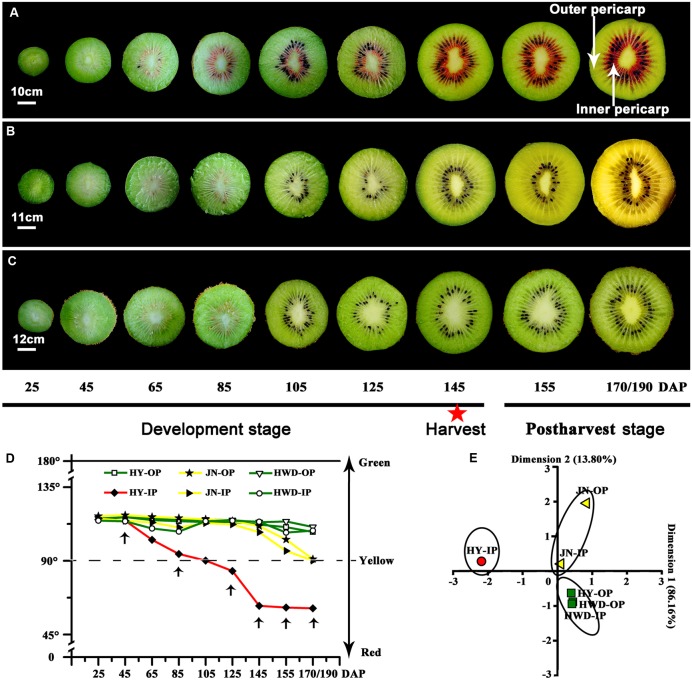
Changes of color in three kiwifruit cultivars. **(A–C)** Bisected fruits of ‘Hongyang’ (HY), ‘Jinnong-2’ (JN) and ‘Hayward’ (HWD) at nine developmental stages. The red star indicates a fruit harvested at 145 DAP (days after pollination). **(D)** Changes of hue angle (*h°*) in the outer and inner pericarps during development and ripening. OP, outer pericarp; IP, inner pericarp; black arrows mark stages were selected for following analyses. **(E)** PCA analyses of hue angles of all samples.

Colors of all three kiwifruit cultivars were green at the beginning of development and with a similar hue angle – both the outer and inner percarps (**Figures 1A–D**). Obvious redness appeared in the inner percarp of ‘Hongyang’ (HY) at 85 DAP, while for its green outer percarp, as well as both outer and inner pericarps of ‘Hayward’ (HWD), remained green throughout, accelerated color change started after 155 DAP in both outer and inner pericarps of ‘Jinnong-2’ (JN) (**Figures 1A–C**). Hue angle decreased dramatically during development in inner pericarp of HY, followed by JN (both outer and inner pericarps), but less so in three green pericarps (**Figure 1D**). A PCA analysis also showed that all outer and inner pericarps of three cultivars were divided into red, yellow, and green groups (**Figure 1E**). Combining the colors of the three cultivars can be described satisfactorily using hue angle which is used in the following analyses.

### Analysis of Pigments in Three Kiwifruit Cultivars

Based on the observations of the phenotypes and on the analyses of hue angles in the fruits of the three kiwifruit cultivars, six stages could be identified for pigment analysis. These stages were S1, S2, S3, S4, and S5 referring to fruits at 45, 85, 125, 145, and 155 DAP, respectively and S6 when fully ripe.

**FIGURE 2:**
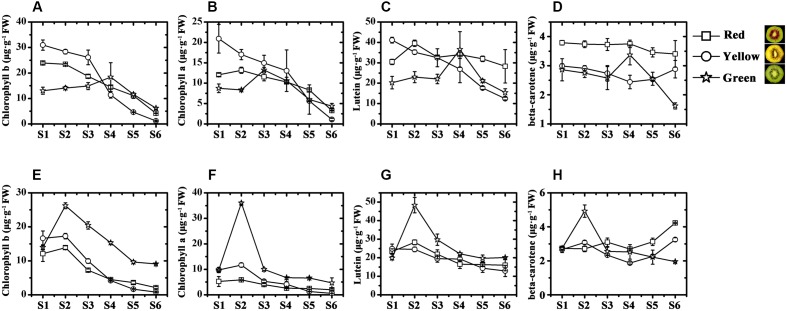
Chlorophyll and carotenoid analyses in ‘Hongyang’ (HY, red), ‘Jinnong-2’ (JN, yellow) and ‘Hayward’ (HWD, green). **(A–D)** Concentrations of chlorophyll b, chlorophyll a, lutein and beta-carotene in the outer and **(E–H)** inner pericarps of the three kiwifruit cultivars during development. Stages S1–S6, represent 45, 85, 125, 145, 155, 170/190 DAP (days after pollination), respectively. Results represent means ± SE of three replicates.

In the outer pericarp tissues of the three kiwifruit cultivars, the chlorophyll b content of JN was higher than that of HY and that of HWD (lowest) before fruits were harvested (**Figure 2A**). But at the last, it was only 1.16 μg⋅g^-1^ FW in JN, significantly lower than that in HY and HWD. So did the contents of chlorophyll a (**Figure 2B**). In the inner pericarp tissues, the contents of both chlorophyll b and chlorophyll a reached peaks at S2 for all three kiwifruit cultivars, followed by a decrease until S6. At this stage the contents were significantly higher in HWD than that in HY and JN (**Figures 2E,F**).

The contents of lutein in the outer pericarp tissues generally decreased slightly but fluctuated somewhat over the development and ripening stages, showing no obvious pattern (**Figure 2C**). It is noteworthy that the content of beta-carotene in HY and HWD generally decreased, but in JN it increased strongly at post-harvest (**Figure 2D**). This change was well correlated with the visible phenotype, indicating beta-carotene seems to be associated with the formation of yellow color in the yellow-fleshed kiwifruit. Further, it was found that in the inner pericarp tissues of all three kiwifruit, the contents of both lutein and beta-carotene in HWD peaked at S2 and then gradually decreased (**Figures 2G,H**). In contrast for HY and JN, the content of lutein showed a slight decrease during development, while the content of beta-carotene increased strongly post-harvest as well as showing change in the outer pericarp of JN.

**FIGURE 3:**
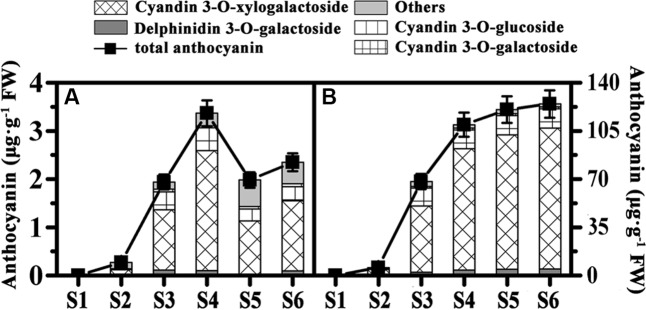
Changes of anthocyanin content of ‘Hongyang’ kiwifruits. **(A)** Outer pericarps. **(B)** Inner pericarps. Results represent means ± SE of three replicates.

To better analyze the pigment differences among the three kiwifruit cultivars, anthocyanins were also detected by HPLC. Unlike carotenoids and chlorophylls detected in all samples, anthocyanins were only detected in the fruit of HY and cyanidin 3-O-xylogalactiside was the major component (**Supplementary Figure S1**). In the fruit of HY, only traces of anthocyanins (or none at all) were detected in the early growth stages in both the inner and outer pericarps of HY (**Figures 3A,B**). In the inner pericarp, it started to increase markedly from S2 until S6, while in the outer pericarp, it changed very slightly – very much lower than in the inner pericarp.

In summary, these results suggest the yellow color of kiwifruit is due not only to the degradation of the chlorophylls but also to the increase in beta-carotene. While the red color of the red-fleshed kiwifruit is due to anthocyanins accumulation.

### Expression Profiles of the Structural Genes Involved in Pigment Metabolism in Kiwifruit

To further investigate what controls the color diversity of green-, yellow-, and red-fleshed kiwifruit, the inner pericarps of the three cultivars were used to analysis the expressions of the key structural genes involved in pigment biosynthesis and degradation using qPCR.

**FIGURE 4:**
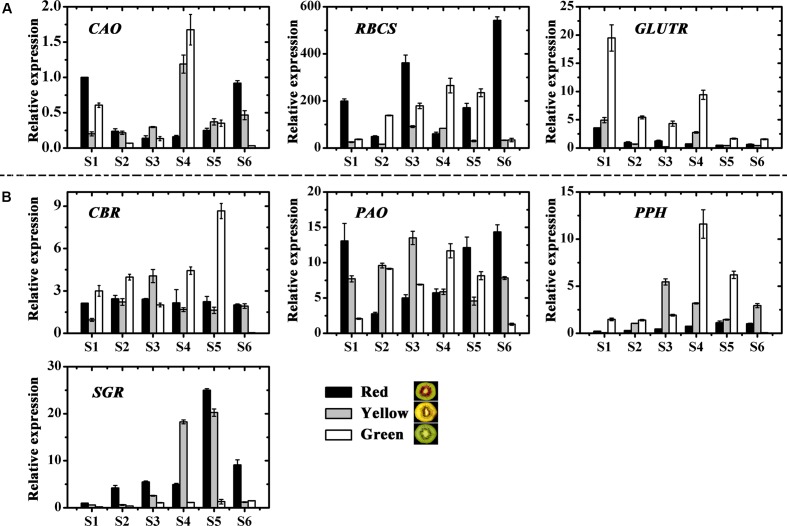
Expression profiles of chlorophyll pathway genes in the inner pericarps of ‘Hongyang’ (HY, red), ‘Jinnong-2’ (JN, yellow) and ‘Hayward’ (HWD, green). **(A)** Biosynthesis genes, **(B)** degradation genes. Error bars are SE for three replicates. *CAO*, Chlorophyll a oxygenase; *CBR*, Chlorophyll b reductase; *GLUTR*, Glutamyl tRNA reductase; *PAO*, Pheophorbide a oxygenase; *PPH*, Pheophytin pheophorbide hydrolase; *RBCS*, Small subunit of ribulose-1,5-bisphosphate Carboxylase; *SGR*, Stay-green.

The genes involved in chlorophyll biosynthesis and degradation were measured (**Figures 4A,B**). *CAO* and *RBCS* expressions varied considerably throughout fruit development in the three kiwifruit cultivars. In contrast, *GLUTR* expression showed higher levels for all stages in the green-fleshed kiwifruit than that in the red- or yellow-fleshed ones. The expression of *CBR* and *PAO* showed no obvious regularity for three kiwifruit cultivars. *PPH* expression in green-fleshed kiwifruit reached a peak, higher than in other two kiwifruit in S4 but except for this peak, overall *PPH* expression was higher in the yellow-fleshed kiwifruit in the later stages. *SGR* expression in red- and yellow-fleshed kiwifruit showed an obvious increase with a peak at S5 that was very much higher than in the green-fleshed kiwifruit.

**FIGURE 5:**
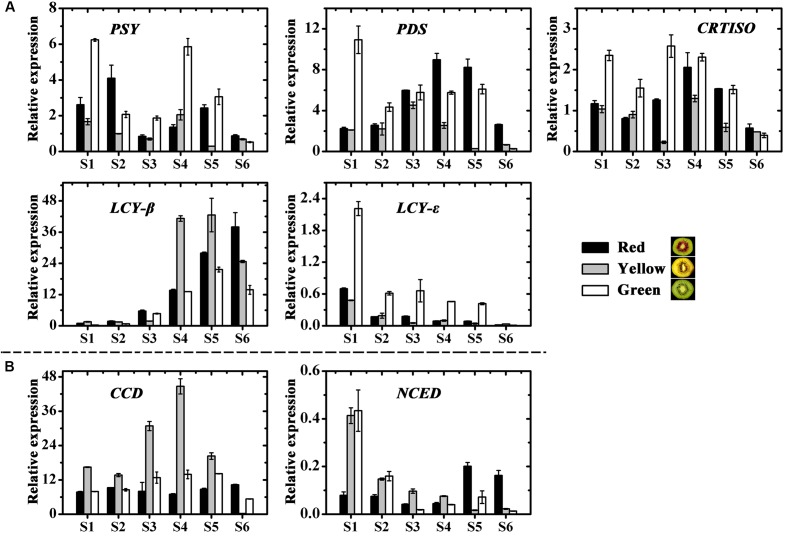
Expression profiles of carotenoid pathway genes in the inner pericarps of ‘Hongyang’ (HY, red), ‘Jinnong-2’ (JN, yellow) and ‘Hayward’ (HWD, green). **(A)** Biosynthesis genes and **(B)** degradation genes. Error bars are SE for three replicates. *PSY*, phytoene synthase; *PDS*, phytoene desaturase; *ZDS*, zeta-carotene desaturase; *CRTISO*, carotene isomerase; *LCY-*β, lycopene beta-cyclase; LCY-𝜀, lycopene epsilon-cyclase; *CCD*, carotenoid cleavage dioxygenease; *NCED*, 9-*cis*-epoxycarotenoid dioxygenase.

Among six carotenoid biosynthesis genes, *ZDS* was not detectable in any samples (data not shown), the relative expression of *PSY*, *PDS*, *CRTISO* and *LCY-*𝜀 in the green-fleshed kiwifruit was higher at most stages than in the other two kiwifruits (**Figure 5A**). While expression of *LCY-*β showed an increase in all three cultivars, the higher levels were measured in the red- and yellow-fleshed kiwifruits than in the green-fleshed one. As for the carotenoid degradation genes *CCD* and *NCED*, both of them showed the highest expression level in the yellow-fleshed fruit at early stages. However, in the final stage, their expressions in yellow-fleshed kiwifruit was significantly lower than in the other two (**Figure 5B**).

**FIGURE 6:**
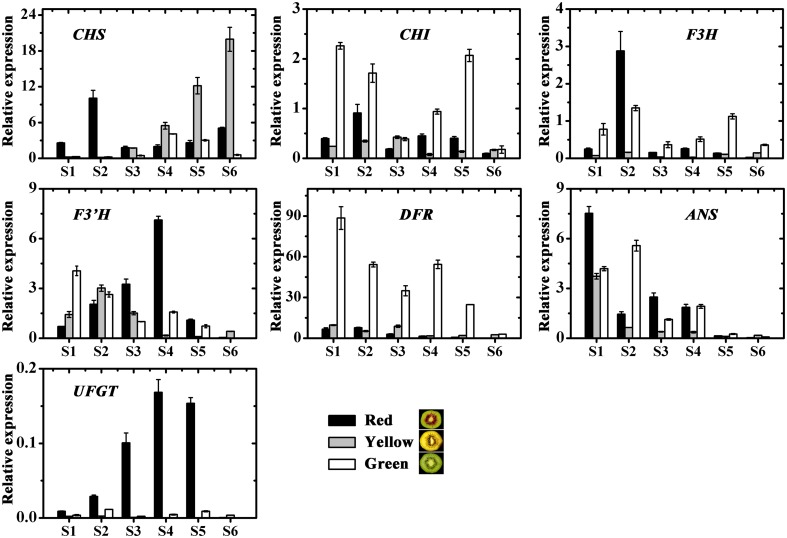
Expression profiles of anthocyanin pathway genes in the inner pericarps of three kiwifruit cultivars ‘Hongyang’ (HY, red), ‘Jinnong-2’ (JN, yellow) and ‘Hayward’ (HWD, green). Error bars are SE for three replicates. *CHI*, Chalcone isomerase; *CHS*, Chalcone synthase; *F3H*, Flavonoid 3-hydroxylase; *F3’H*, Flavonoid 3’-hydroxylase; *DFR*, Dihydroflavonol 4-reductase; *ANS*, Anthocyanidin synthase; *UFGT*, UDP flavonoid glycosyltransferases.

The expression profiles of the anthocyanin biosynthetic pathway genes, including *CHS*, *CHI*, *F3H*, *F3′H*, *DFR*, *ANS* and *UDP flavonoid glycosyltransferases (UFGT)*, were investigated in three kiwifruit during fruit development (**Figure 6**). Apart from *UFGT*, most of the others, were expressed in both the red and also in the non-red tissues. In contrast, *UFGT* was highly expressed in the red inner pericarps but not in the green or yellow inner pericarps.

### Correlation Analysis

To find significant statistical correlations between gene expression and flesh color, the Pearson’s correlation coefficient (r) test was used with the above qPCR data, pigment contents and hue angles. *SGR* showed a significant positive correlation with the degradation of chlorophylls (Supplementary Table S3), and *LCY-*β correlated well with the beta-carotene and hue angles (Supplementary Table S4). Also, *UFGT* showed a significant negative correlation with hue angle and a significant positive correlation with anthocyanin concentration (Supplementary Table S5). Apart from the genes identified above, none of the others showed significant correlations with flesh color.

### Expression Profiles of Anthocyanin Transcription Factors in Different Colored Kiwifruits

**FIGURE 7:**
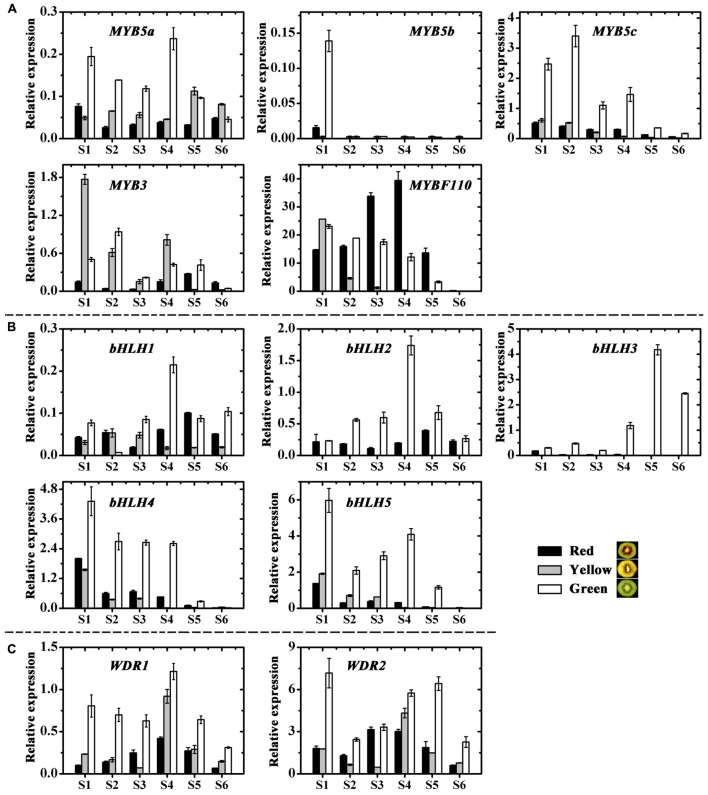
Expression profiles of anthocyanin transcription factors in the inner pericarps of ‘Hongyang’ (HY, red), ‘Jinnong-2’ (JN, yellow) and ‘Hayward’ (HWD, green). **(A)** MYBs, **(B)** bHLHs and **(C)** WD40s. Error bars are SE for three replicates.

Structural plant genes are usually regulated by transcription factors, but we found no transcription factors regulating the chlorophylls and carotenoids from the genome of kiwifruit^2^ based on previous reports. While twelve anthocyanin transcription factors, including five MYBs, five bHLHs and two WD40s, were screened in kiwifruit and named according to the phylogenetic analysis (**Supplementary Figure S2**) (Hichri et al., 2010; An et al., 2012; Liu et al., 2013b; Schaart et al., 2013; Tuan et al., 2015; Lai et al., 2016). The expression profiles of these kiwifruit transcription factors were then examined by real-time qPCR (**Figures 7A–C**).

Except for the first stage, *MYB5b* was either not expressed at all or showed extremely low expression levels for all samples at all stages of fruit development (**Figure 7A**). In contrast, *MYB5a*, *MYB5c* and *MYB3* were expressed in all samples and showed higher expressions in green and yellow fruits than in red fruits. Overall, the variations of these four MYBs were correlated neither with anthocyanin content nor with the expression of *UFGT* (Supplementary Table S6). However, the transcript level of *MYBF110* in all three fruits showed significant correlations with both anthocyanin content (|*r*| = 0.550) and with the expression of *UFGT* (|*r*| = 0.609). Especially during the coloring stages (S2 to S4), its expression increased and reached maximum value at S4 in the red fruit, while for the green and yellow fruits, it decreased, showing significantly lower levels than in the red fruit (**Figure 7A**). These findings suggest an essential role for *AcMYBF110* in the anthocyanin biosynthesis of red-fleshed kiwifruit.

Of the five *bHLH* genes, all showed much higher expressions in the green fruit than in the red and yellow ones at all stages of development (**Figure 7B**). For *bHLH1*, *bHLH2* and *bHLH3*, it increased until S4, at which stage expression reached a maximum and then decreased in the later stages. For *bHLH4* and *bHLH5*, expression fluctuated in the early stages but showed an overall decreasing trend during fruit development. All five genes showed no obvious correlation with anthocyanin biosynthesis (Supplementary Table S6).

Lastly, the expression of both *WDR1* and *WDR2* was significantly higher in green fruit than in red and yellow fruits at all stages (**Figure 7C**), being not consistent with anthocyanin content and *UFGT* expression (Supplementary Table S6), although it showed a tendency to increase in red fruit at the coloring stages.

### Sequence and Expression Analysis of *AcMYBF110*

**FIGURE 8:**
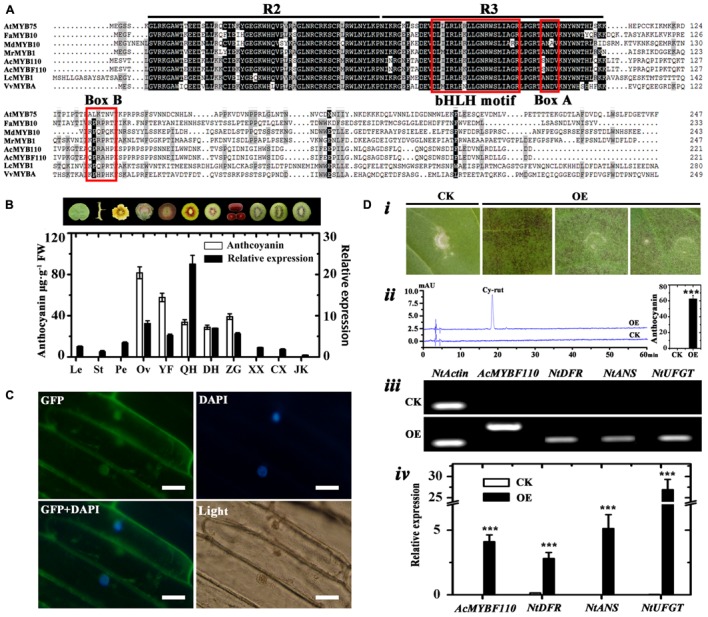
Analyses of *AcMYBF110*. **(A)** Amino acid sequence alignment of AcMYBF110 and other plant anthocyanin-promoting MYBs. The R2 and R3 MYB motifs are indicated. The bHLH motif indicates residues needed for the interaction with the bHLH partner, box A and box B are well-conserved in anthocyanin-promoting MYBs. **(B)** Anthocyanin contents and expression profiles of *AcMYBF110* in various tissues of ‘Hongyang’ and fruit cultivars. Le, leaves; St, stems; Pe, petals; Ov, ovary; YF, young fruits (10 days after pollination); QH, ‘Qihong’; DH, ‘Dohong’; ZG, *Actinidia arguta* var. purpurea; XX, ‘Xuxiang’; CX, ‘Cuixiang’; JK, Jinkui’. Data represent means ± SE of three replicates. **(C)** Subcellular localization of AcMYBF110 in *Nicotiana benthamiana* leaves. Scale bars: 50 μm. **(D)** Transient assays demonstrate the function of *AcMYBF110* as a regulator of anthocyanin biosynthesis. ***(i)*** Assay leaves of *AcMYBF110* exhibited anthocyanin accumulation. ***(ii)*** HPLC analysis of leaves injected with empty vector (CK) and 35S:AcMYBF110-GFP (OE). ***(iii*,*iv)*** Expression analysis of *AcMYBF110* and key anthocyanin biosynthesis genes in CK and OE leaves. Data were analyzed with *t*-test. ^∗^*P* < 0.05; ^∗∗^*P* < 0.01; ^∗∗∗^*P* < 0.001.

The full-length cDNA of *AcMYBF110* was cloned from ‘Hongyang’ fruit. The predicted AcMYBF110 protein contained a highly conserved N-terminal R2R3 repeat of a DNA-binding domain with a bHLH motif (Zimmermann et al., 2004). Second, the ANDV motif (Box A) and the [R/K]Px[P/A/R]xx[F/Y] motif (Box B) are present in AcMYBF110 (**Figure 8A**). Both of these motifs are well conserved in anthocyanin-promoting MYBs (Kranz et al., 1998; Lin-Wang et al., 2010). AcMYBF110 had a high degree of homology with other plant anthocyanin-promoting MYBs (**Figure 8A**). For example, the amino acids in the R2R3 DNA-binding domain share 95.15% identity to AcMYB110, 83.50% identity to LcMYB1, 82.52% identity to FaMYB10, and 81.30% identity to MrMYB1.

To examine whether the expression patterns of *AcMYBF110* coincided spatially or inter-varietally with anthocyanin accumulation in kiwifruit, real-time qPCR was used to investigate its expression levels in different tissues of ‘Hongyang’ and in the other cultivars. The results showed significantly higher levels of expression were found in two red tissues and in the three red-fleshed cultivars (**Figure 8B**). These correlated well with the high anthocyanin contents of these samples. In contrast, lower levels were observed in the other green tissues in which anthocyanins were barely detectable. These findings further confirm that *AcMYBF110* may be involved in regulating anthocyanin biosynthesis in kiwifruit.

### Subcellular Localization Analysis of AcMYBF110

To determine the subcellular localization of the putative protein encoded by AcMYBF110, the transient expression assay of this protein fused to the green fluorescent protein (GFP) was carried out in 6-week-old *N. benthamiana* leaves. As shown in **Figure 8C**, the AcMYBF110-GFP fusion protein signal was observed both in the nucleus and cytoplasm. This suggests the AcMYBF110 protein could act as a transcriptional regulator in plant cells.

### Functional Test of *AcMYBF110* in Tobacco by Transient Overexpression

To further confirm the regulation role of *AcMYBF110* in anthocyanin biosynthesis, the transcriptional activity of *AcMYBF110* was tested using a tobacco transient color assay. The above fused construct under the action of 35S (35S:AcMYBF110-GFP) was syringe-infiltrated into the abaxial surfaces of expanding *N. tabacum* leaves. The empty vector (35S:GFP) served as a control. An intense red pigmentation was observed at the infiltration sites 6 days after being transformed with 35S:AcMYBF110-GFP (**Figure 8D*i***), while the infiltration sites remained green in leaves transformed with 35S:GFP. Anthocyanin extraction and HPLC analysis showed the leaves transformed with 35S:AcMYBF110-GFP contained anthocyanin but not those with 35S:GFP (**Figure 8D*ii***). Moreover, *AcMYBF110* was highly expressed in leaves transformed with 35S:AcMYBF110-GFP but no transcript was detected in leaves transformed with 35S:GFP (**Figures 8D*iii*,*iv***). We then carried out qPCR analyses of the expression of key anthocyanin synthesis genes in tobacco leaves, including *NtDFR*, *NtANS* and *NtUFGT*. The expression levels of these were higher in the leaves transformed with 35S:AcMYBF110-GFP than in those transformed with 35S:GFP by 17.73-, 2185.20-, and 801.58-times, respectively.

## Discussion

### Pigment Changes in Green-, Yellow-, and Red-Fleshed Kiwifruit

Chlorophyll, carotenoid and anthocyanin are the three most important color pigments in plant tissues, including in fruit. We measured the concentrations of these three pigments in the outer and inner pericarps of three different-colored kiwifruits at various developmental stages. The concentrations of chlorophyll b and chlorophyll a showed similar variation trends in all kiwifruits (**Figures 2A,B,E,F**) but were significantly lower in the yellow fruits (JN) than that in the red (HY) or green (HWD) fruits when fully ripe. Meanwhile, the beta-carotene content decreased slightly in the early stages in all three cultivars. Subsequently, beta-carotene increased significantly until S6 in JN but remained low in HY and HWD (**Figures 2C,D,G,H**). In contrast, there was no obvious pattern for lutein content, which agrees with Ampomah-Dwamena et al. (2009). These results indicate that the formation of yellowness in kiwifruit is related not only to a degradation of chlorophylls but also to an increase in beta-carotene (Ampomah-Dwamena et al., 2009; Pilkington et al., 2012). Anthocyanin was detected only in HY fruits with very low or no detectable levels found in the green outer pericarps from S1 to S6 of and green inner pericarp at S1, while higher levels were detected in the red inner pericarps from S3 to S6 (**Figure 3**). This indicates anthocyanin is the pigment primarily responsible for the formation of the red color in the inner pericarp of HY (Li et al., 2015; Man et al., 2015).

### Expression of Pigment Biosynthesis and Degradation Genes in Kiwifruit

Chlorophyll de-greening is a coordinated process. It seems to be involved with a down-regulation of chlorophyll biosynthesis and an up-regulation of chlorophyll degradation. SGR proteins have been shown previously to play a critical role in the initiation of chlorophyll degradation and senescence (Luo et al., 2013; Sakuraba et al., 2015). We measured the transcript levels of the chlorophyll biosynthesis and degradation genes and found the genes are expressed in all three cultivars (**Figure 4**). Based on correlation analysis between gene expression and hue angles and chlorophyll content, SGR showed a significant positive correlation with the degradation of chlorophylls (Supplementary Table S3). This is in agreement with results obtained by Pilkington et al. (2012), who showed *SGR2* was a potential regulatory step of chlorophyll degradation. To date, the relationship between carotenoid gene expression and carotenoid accumulation has been investigated in a range of crop species. For example, *PDS* was found to contribute directly to beta-carotene accumulation in citrus (Fanciullino et al., 2008). Ha et al. (2007) suggest that high concentrations of carotenoids are associated with high transcription levels of *PDS* and *PSY* in pepper. Meanwhile, the expression level of *PSY* was found to be higher in high carotenoid tomatoes, compared with low carotenoid ones (Morris et al., 2004). In our study, only the transcription level of *LCY-*β was well correlated with beta-carotene contents and hue angles (**Figure 5** and Supplementary Table S4). This indicates that *LCY-*β may be a key determinant of beta-carotene biosynthesis in kiwifruit, suggesting that *LCY-*β is involved in controlling and regulating the yellow color formation in kiwifruit (Ampomah-Dwamena et al., 2009). These results suggest carotenoid biosynthesis may be differently controlled in different species. All the above results suggest that yellow color formation may be controlled by *SGR* together with *LCY-*β. During fruit development and ripening, *SGR* activates the degradation of chlorophyll, meanwhile *LCY-*β catalyzes beta-carotene biosynthesis. Together these cause flesh color to change from green to yellow.

It has been shown UFGT enzyme is key to anthocyanin biosynthesis in peach (Tuan et al., 2015), pear (Wang et al., 2013), strawberry (Gonzalez et al., 2008). While for apple, most stuctural genes in the anthocyanin pathway function in concert to determine fruit anthocyanin level (El-Sharkawy et al., 2015). In the present study, we measured and compared the expression levels of *CHS*, *CHI*, *F3H*, *F3′H*, *DFR*, *ANS* and *UFGT* in the green-, yellow-, and red-inner pericarps of the three cultivars during development (**Figure 6**). Apart from *UFGT*, most of the others, were expressed in both the red and also in the non-red tissues, showing a weak correlation with anthocyanin content (**Figure 6** and Supplementary Table S5). In contrast, *UFGT* showed the strongest correlation with anthocyanin content, being highly expressed in the red inner pericarps but not in the green or yellow inner pericarps. This agrees results obtained by Montefiori et al. (2011), who suggests the red inner pericarps of ‘HD22’ is the result of high expressions of *AcF3GT1*.

### Expression of Transcription Factors Involved in Anthocyanin Biosynthesis in Kiwifruit

In plants, the structural genes are always regulated by transcription factors. There are, however, few studies of transcription factors regulating the genes of chlorophyll and carotenoid biosynthesis and degradation. Those studies that exist are mostly in model plants (Tang et al., 2012; Liu et al., 2013a). Hence, no transcription factors regulating chlorophylls and carotenoids have been screened in our study. More importantly, anthocyanin content was the most significant difference between the three kiwifruit cultivars in this study. Therefore, our study only analyzed the anthocyanin transcription factors in kiwifruit. A growing body of evidence suggests anthocyanin biosynthesis in plants is controlled by a transcription complex, composed of two transcription factors belonging to the R2R3-MYB and the bHLH-MYC protein families. A WD40 co-factor protein is also involved. These three proteins activate the expression of a downstream structural gene in the anthocyanin pathway by forming the MYB-bHLH-WD40 (MBW) complex (Schaart et al., 2013; Xu et al., 2014, 2015).

At this stage, the MYBs determining anthocyanin biosynthesis have been well characterized in many species (Chagne et al., 2013; Perez-Diaz et al., 2016; Zhai et al., 2016). In our study, we used the above anthocyanin regulation related MYBs to screen the genome of kiwifruit^3^. We found five MYBs and named these: *AcMYBF110*, *AcMYB5a*, *AcMYB5b*, *AcMYB5c* and *AcMYB3* based on the names of similar genes (**Supplementary Figure S2A**). Expression and correlation analyses showed that transcript of *AcMYBF110* was significantly correlated with both anthocyanin content and also with the expression of UFGT (**Figure 7A** and Supplementary Table S6). Similar analyses for the other MYBs showed much weaker correlations. This indicates *AcMYBF110* may plays a crucial role in regulating anthocyanin biosynthesis in kiwifruit. Therefore, this was cloned from fruit of ‘Hongyang’ and its expression levels were found to be correlated with anthocyanin contents (**Figures 8A,B**). Moreover, the subcellular localization analysis showed that AcMYBF110 is located both in the nucleus and the cytoplasm. This suggests that the AcMYBF110 protein is the transcriptional regulator in these plant cells (**Figure 8C**). Further, transient color assays showed that, as with peach *PpMYB10.1* (Zhou et al., 2015) and *Actinidia chinensis* *AcMYB110* (Montefiori et al., 2015), *AcMYBF110* can autonomously induce anthocyanin accumulation in *N. tabacum* leaves by activating transcription of *NtDFR*, *NtANS* and *NtUFGT* (**Figure 8D**). This differs from apple *MdMYB10* and *MdMYB110* which induce anthocyanin accumulation by necessarily interacting with *bHLH3* (Chagne et al., 2013). These results confirm *AcMYBF110* is a key R2R3 MYB transcription factor regulating anthocyanin biosynthesis in red-fleshed kiwifruit. Further studies are required to clarify which anthocyanin structural genes are regulated by *AcMYBF110*.

The bHLH proteins function as anthocyanin regulators and have been reported in model plants and fruit species including: maize (Ludwig et al., 1989), *Arabidopsis* (Zhang et al., 2003), grape (Hichri et al., 2010), peach (Rahim et al., 2014) and litchi (Lai et al., 2016). They usually serve as co-factors interacting with the R2R3 MYBs to induce anthocyanin biosynthesis. In this study, we found five bHLHs from the genome of ‘Hongyang,’ and their expression levels were measured by qPCR (**Figure 7B**). All five bHLHs were much more highly expressed in green fruit than in red or yellow fruit at all stages of development. Moreover, they showed no significant correlation with anthocyanin accumulation (Supplementary Table S6). This indicates that, as in apple (Espley et al., 2007), peach (Rahim et al., 2014) and litchi (Lai et al., 2016), bHLHs likely depends on co-expression with other regulatory factors (such as R2R3 anthocyanin-promoting MYBs) to induce anthocyanin biosynthesis in the red-fleshed kiwifruit. This instead of contributing directly to anthocyanin biosynthesis. However, in most plants more than one bHLH factor controls anthocyanin biosynthesis and other metabolic pathways. An increasing body of evidence supports the view that the different bHLHs have specific functions within each species. Hence, it is worth further study to ascertain which of the bHLH serves as a partner to *AcMYBF110* so as to regulate coloration in the red-fleshed kiwifruit.

The WD40 group is members of the MBW transcription complex. This group is related to anthocyanin biosynthesis and has been isolated in a number of model plant species. Initially, it included *AN11* which controls flower pigmentation in petunia (de Vetten et al., 1997) and *AtTTG1* in *Arabidopsis* (Walker et al., 1999). More recently, WD40 members involved in anthocyanin biosynthesis have been isolated in a number of fruit species (Brueggemann et al., 2010; Matus et al., 2010; Schaart et al., 2013). Based on these WD40s, we found two WD40 members (*WDR1* and *WDR2*) in kiwifruit (**Supplementary Figure S2C**). Analysis of qPCR showed the expression of *WDR1* was significant higher in green fruit than in red or yellow fruits at all stages. It also showed a trend for increase in the red fruit in the coloring stages. Similar profiles were found for the expression of *WDR2* (**Figure 7C**). Overall, the expressions of *WDR1* and *WDR2* were not consistent with anthocyanin content and *UFGT* expression in the three different colored kiwifruits examined here (Supplementary Table S6). However, if analyzed only in the red fruit, their expressions were highly correlated with both anthocyanin content and with *UFGT* expression (data not shown). These findings suggest these WDRs are likely to play different roles in different kiwifruit cultivars. For example, in red kiwifruit they are primarily involved in regulation of anthocyanin biosynthesis, while in the green and yellow kiwifruits, their involvement seems mainly to be the regulation of other physiological and biochemical processes. In other plants, they have been shown to be involved in defense and response to various biotic and abiotic stresses (Lee et al., 2010; Chen and Brandizzi, 2012).

## Conclusion

Our analysis of pigments changes and expression differences of pathway genes in different colored kiwifruits suggests the formation of yellowness in kiwifruit is due to the degradation of chlorophylls and also to an increase in beta-carotene, under the control of *SGR* and *LCY-*β. Meanwhile, the red color formation is due to rising anthocyanin content controlled by UFGT. The expression of transcription factors suggests that *AcMYBF110* is a crucial MYB regulating anthocyanin biosynthesis. *AcMYBF110* was expressed at a significantly higher level in red tissues and cultivars than that in green or yellow tissues or cultivars. Subcellular localization analyses show that AcMYBF110 is located both in the nucleus and the cytoplasm. This suggests the AcMYBF110 protein could act as transcriptional regulator in plant cells. Further, a transient color assay revealed that *AcMYBF110* could autonomously induce the anthocyanin accumulation in *N. tabacum* leaves by activating the transcription of *NtDFR*, *NtANS* and *NtUFGT*. For bHLHs and WD40s, expression differences show they may depend on *AcMYBF110* forming a MBW complex to regulate anthocyanin biosynthesis, rather than having a direct effect. The structural components of this MBW complex and the mechanisms regulating of anthocyanin biosynthesis in kiwifruit remain to be explored.

## Author Contributions

YL designed the experiments, performed the research, wrote and revised this manuscript. BZ, YQ, XC, and CL performed experiments and helped revised manuscript. ZL designed the experiments and provided all samples tested. XR designed the experiments, discussed results, and revised this manuscript. All authors have participated in this research and approved the final manuscript.

## Conflict of Interest Statement

The authors declare that the research was conducted in the absence of any commercial or financial relationships that could be construed as a potential conflict of interest.

## Footnotes

1 http://bioinfo.bti.cornell.edu/kiwi

2 http://bioinfo.bti.cornell.edu/kiwi

3 http://bioinfo.bti.cornell.edu/kiwi

## Supplementary Material

FIGURE S1 HPLC chromatograms of anthocyanins from outer (OP) and inner (IP) pericarps of ‘Hongyang’ (HY), ‘Jinnong-2’ (JN) and ‘Hayward’ (HWD) at 520 nm. (1) Delphinidin 3-O-galactoside; (2) unknown composition; (3) cyanidin 3-O-xylogalactoside; (4) cyanidin-galactoside; (5) cyanidin 3-O-xylogalactoside; (6) cyanidin 3-O-glucoside; (7) cyanidin-pentoside; (8) unknown composition.

FIGURE S2 Phylogenetic analyses of selected plants anthocyanin regulating transcription factors and putative kiwifruit factors. **(A)** MYBs, **(B)** bHLHs and **(C)** WD40s.
