# Supplementary figures and images for: Expression Differences of Pigment Structural Genes and Transcription Factors Explain Flesh Coloration in Three Contrasting Kiwifruit Cultivars
Source: Front Plant Sci. 2017 Sep 1;8:1507. doi: 10.3389/fpls.2017.01507 (PMC5586210; doi:10.3389/fpls.2017.01507)

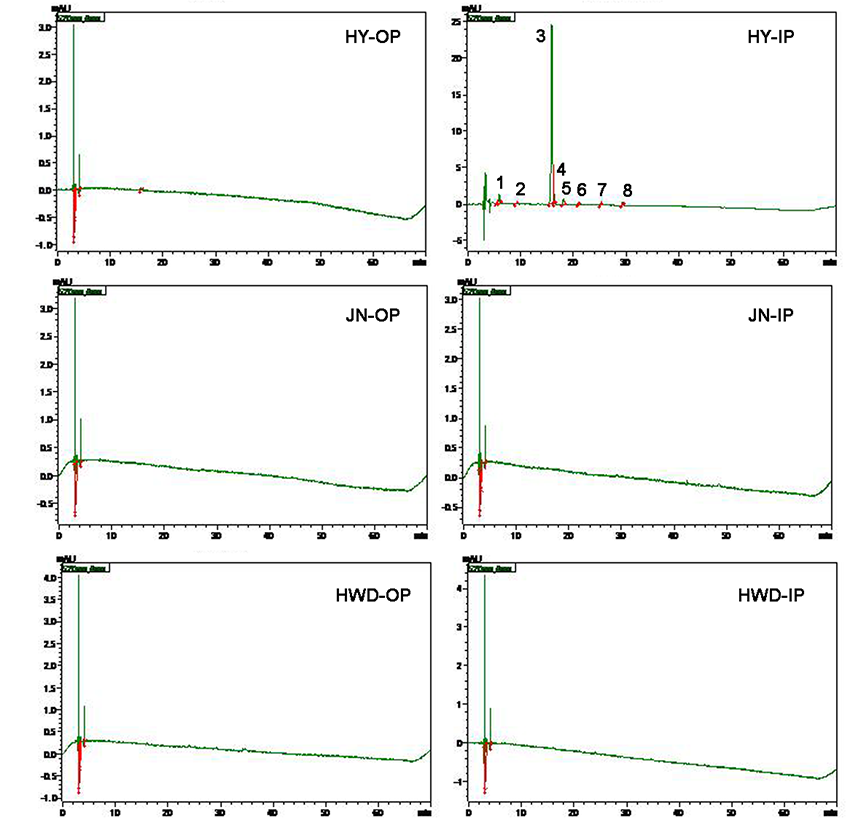

Supplement: FIGURE S1 — HPLC chromatograms of anthocyanins from outer (OP) and inner (IP) pericarps of ‘Hongyang’ (HY), ‘Jinnong-2’ (JN) and ‘Hayward’ (HWD) at 520 nm. (1) Delphinidin 3-O-galactoside; (2) unknown composition; (3) cyanidin 3-O-xylogalactoside; (4) cyanidin-galactoside; (5) cyanidin 3-O-xylogalactoside; (6) cyanidin 3-O-glucoside; (7) cyanidin-pentoside; (8) unknown composition. [file Image_1.TIF]

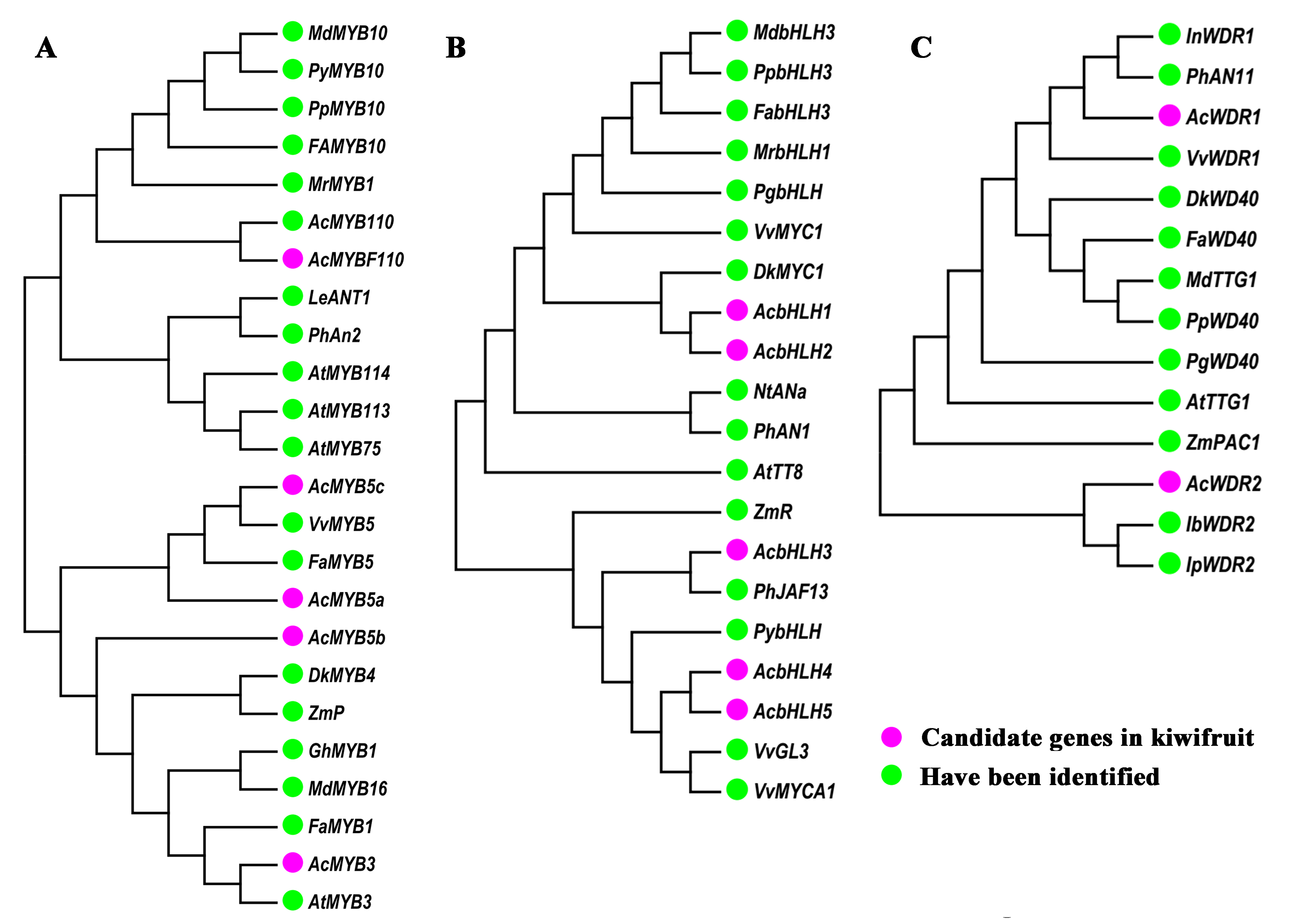

Supplement: FIGURE S2 — Phylogenetic analyses of selected plants anthocyanin regulating transcription factors and putative kiwifruit factors. (A) MYBs, (B) bHLHs and (C) WD40s. [file Image_2.TIF]
